# Intrinsic capacity and quality of life trajectories in older Europeans by sex and region

**DOI:** 10.21203/rs.3.rs-9407485/v1

**Published:** 2026-05-04

**Authors:** Rafael Llorens-Ortega, Carmen Bertran-Noguer, Dolors Juvinyà-Canal, Josep Garre-Olmo, Cristina Bosch-Farré

**Affiliations:** EUIT University Center, Autonomous University of Barcelona (UAB); University of Girona; University of Girona; University of Girona; University of Girona

**Keywords:** intrinsic capacity, quality of life, aging, sex differences, social determinants of health, SHARE

## Abstract

Understanding determinants of quality of life (QoL) in older adults is crucial in aging societies. Intrinsic capacity (IC), combining physical and mental capacities, may influence QoL changes, but evidence on specific IC domains and QoL is limited. This study examines associations between IC domains and two-year QoL changes in older Europeans, focusing on sex and regional differences.

**Methods:**

Data from 11,493 adults aged ≥50 from 13 European countries in SHARE Waves 5 and 6 (2013–2015) were analyzed. IC was operationalized across five domains: mobility, cognition, psychological well-being, sensory function, and vitality. Exploratory factor analysis validated IC’s multidimensional structure. Network analysis assessed domain interrelations and links to QoL (CASP-12). Sex and regional differences were explored via stratified analyses and ANOVA.

**Results:**

IC domains formed a coherent multidimensional construct. Psychological well-being and mobility showed the strongest associations with QoL. Depressive symptoms and fatigue correlated negatively with CASP-12 (r = −0.284 and −0.324, p < 0.001). Cognitive and mobility domains had weaker but significant links. Over two years, modest IC declines paralleled QoL changes. Women and individuals in Southern and Eastern Europe exhibited greater IC deficits and lower QoL.

**Conclusions:**

Intrinsic capacity significantly influences short-term QoL changes in older Europeans. Psychological and mobility domains are key targets for interventions. Addressing sex and regional disparities in IC may improve well-being and reduce inequalities in aging populations.

## Introduction

Population aging is a global phenomenon that is profoundly impacting countries worldwide, particularly those with higher levels of development, due to increased life expectancy and declining fertility rates (WHO, 2024). Demographic projections estimate that the proportion of individuals aged 65 and older will rise from 9.7% in 2023 to 16.4% by 2050, with Europe being one of the most affected regions (WHO, 2024). This accelerated demographic shift presents significant challenges for public health and social systems, increasing the demand for strategies that promote healthy aging and enhance the quality of life (QoL) of older adults (United Nations, 2021). Quality of life is widely recognized as a multidimensional construct influenced not only by health status but also by social participation, autonomy, and broader societal conditions (McGregor et al., 2009; Galloway, 2006).

In response to this challenge, the World Health Organization (WHO) has proposed a model of healthy aging that emphasizes the importance of functional ability, defined as the dynamic interaction between an individual’s intrinsic capacity and their environment (WHO, 2015). This model recognizes that aging is shaped not only by biological factors but also by psychosocial and environmental determinants (WHO, 2020). Within this framework, intrinsic capacity (IC), conceptualized by the WHO as the composite of an individual’s physical and mental capacities, plays a pivotal role in shaping trajectories of quality of life (QoL) in older adults, representing a paradigm shift from disease-centered models to a function-centered approach in healthy aging (WHO, 2015). IC is operationalized through five key domains as proposed in the WHO ICOPE framework: mobility, cognitive capacity, sensory function, psychological well-being, and vitality (WHO, 2019).

Recent research has demonstrated that assessing IC offers a more comprehensive and nuanced understanding of healthy aging and its implications for QoL (Angelsen et al., 2024; Chhetri, 2022). Nonetheless, challenges remain in the development and validation of large-scale tools to measure these domains reliably (Rojano et al., 2023). Promoting active and healthy aging requires targeted interventions aimed at preserving and enhancing these capacities, thereby supporting physical, psychological, and social well-being. Social participation has also been identified as an important determinant of well-being and functional outcomes in later life, contributing to both health and quality-of-life trajectories (Oshio et al., 2024). Studies such as that by Takeda et al. (2024) have highlighted that improvements in socioeconomic conditions and access to healthcare can help maintain IC even in the presence of chronic conditions (Takeda et al., 2024). Furthermore, active social participation has been positively associated with higher IC and improved QoL, underscoring the need to reduce social isolation and strengthen support networks (López-Ortiz et al., 2022).

Sex disparities in aging and their impact on QoL remain a critical area of inquiry. Recent studies have identified persistent sex inequalities in health, education, and QoL among older adults (Ahrenfeldt & Möller, 2021; Salinas-Rodríguez et al., 2024). Older women are more likely to experience poorer health outcomes and lower QoL compared to men (Llorens-Ortega et al., 2024). In this context, IC plays a pivotal role in shaping QoL trajectories, justifying a sex-sensitive analytical approach.

The Survey of Health, Ageing and Retirement in Europe (SHARE) is one of the most comprehensive longitudinal databases available, offering valuable insights into how social, economic, and health-related factors influence QoL among older adults across diverse European contexts (Börsch-Supan et al., 2013). Its multidisciplinary and longitudinal design enables the exploration of the interplay between social determinants of health, IC, and other relevant variables in shaping QoL outcomes.

It is essential to recognize that intrinsic capacity does not evolve in isolation, but is strongly shaped by social determinants of health, including education, socioeconomic status, living conditions, and access to healthcare and social support (Marmot, 2020; WHO, 2015). These structural factors influence both the preservation and decline of functional domains, and their unequal distribution contributes to disparities in aging outcomes. Evidence consistently shows that women, while living longer, are more likely to experience multimorbidity, functional limitations, and poorer self-rated health than men (Crimmins et al., 2019; Oksuzyan et al., 2019). Similarly, cross-national studies demonstrate that older adults in Southern and Eastern Europe, where welfare systems are less comprehensive and socioeconomic inequalities more pronounced, face a higher risk of functional decline and reduced quality of life compared to those in Northern and Continental Europe (Börsch-Supan et al., 2013; Llorens-Ortega et al., 2024). Therefore, given that IC encompasses key functional domains, its preservation is hypothesized to directly influence trajectories of QoL in older adults. We hypothesize that declines in intrinsic capacity domains will predict deteriorations in QoL over two years, with women and residents of Southern and Eastern Europe experiencing disproportionately greater declines.

By integrating functional domains of intrinsic capacity with social determinants of health in a cross-national European sample, this study contributes to the growing literature examining how biological, psychological, and social factors jointly shape quality-of-life trajectories in later life. Although limited to two waves, this longitudinal design allows examination of short-term change patterns in intrinsic capacity and their association with QoL dynamics.

## Materials and Methods

### Study Design

This study is a prospective, analytical cohort study based on population-level data from the Survey of Health, Ageing and Retirement in Europe (SHARE) (Börsch-Supan et al., 2013; Malter, 2015). SHARE is a multinational longitudinal study that investigates health, socioeconomic, and demographic factors among non-institutionalized adults aged 50 and older. Participants are interviewed biennially, enabling international comparisons and longitudinal analyses of aging-related factors. To minimize bias SHARE employs standardized data collection procedures, including harmonized questionnaires and probabilistic representative sampling to ensure representativeness across countries.

Data for this study were drawn from Waves 5 (2013) and 6 (2015), which provide the most consistent and comparable measures of the intrinsic capacity domains across countries and allow for a robust longitudinal design. Although more recent waves are available, key variables were not fully harmonized in later datasets, and attrition was higher compared with the 2013–2015 interval, reducing response rates and completeness of variables of interest which could bias longitudinal analyses.

### Study Population

In Wave 5 (2013), a total of 59,421 individuals were surveyed across 13 European countries: Germany, Austria, Belgium, Denmark, Slovenia, Spain, Estonia, France, Italy, Luxembourg, Sweden, Switzerland, and the Czech Republic. Inclusion criteria for this study required participants to be aged 50 or older, reside in one of the 13 selected countries, consent to participate, have taken part in both consecutive waves under analysis, possess complete data for all variables of interest, and not be institutionalized at the time of the interviews.

A total of 11,493 individuals met these criteria. The remaining participants were excluded due to attrition, death, non-participation in the subsequent wave, or missing data in key variables.

**Figure 1:**
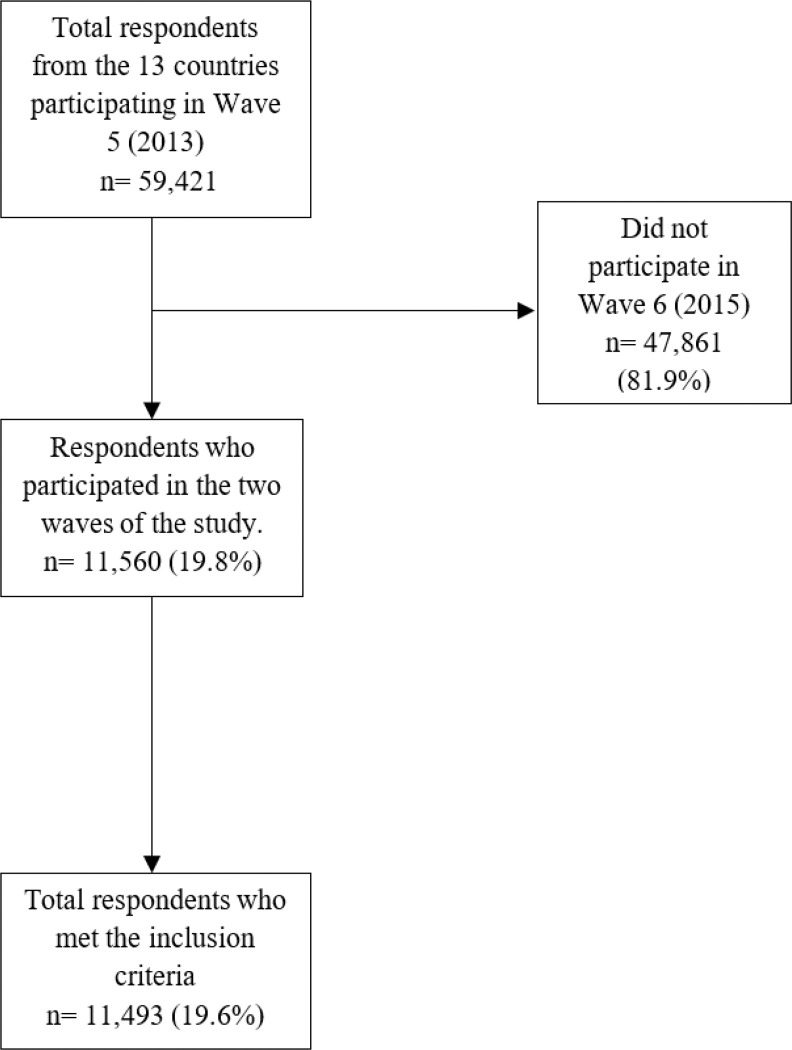
Flowchart of the sample selection process.

Figure 1 presents the flow diagram detailing the total number of respondents in Wave 5, exclusions due to non-participation or missing data, and the final analytical sample.

To assess potential bias arising from non-participation in Wave 6, a comparative analysis was conducted using Propensity Score Matching (PSM). Participants and non-participants were matched based on key sociodemographic variables such as sex, age, and geographic region. This approach ensured that the analytical sample was representative of the general Wave 5 population, with only minor differences in age and a slightly lower proportion of women. Quality of life scores (CASP-12) were compared between matched groups, revealing no statistically significant differences. The participant selection methodology is detailed in a previous study (Llorens-Ortega et al., 2024) and is available for download via GitHub (Vila, J., 2024). Supplementary details of this analysis are provided in **Table S1.**

For the analysis of regional differences in the evolution of QoL and IC, countries were grouped into four regional clusters based on the 2023 Eurostat report on welfare models in Europe (European Commission, 2023):

1. Northern Europe: Denmark and Sweden (Social Democratic regimes)
2. Continental Europe: Austria, Germany, Belgium, France, Luxembourg, and Switzerland (Corporatist regimes)
3. Southern Europe: Spain and Italy (Southern European regimes)
4. Eastern Europe: Slovenia, Estonia, and the Czech Republic (Post-Socialist regimes)

This grouping was selected to reflect welfare regime typologies that influence social and health policies, although some heterogeneity within clusters may exist.

### Data Collection Procedures

Data were collected through Computer-Assisted Personal Interviews (CAPI), with an average duration of 90 minutes. Interviews were conducted in participants’ homes using standardized questionnaires covering a wide range of topics, including IC domains, social determinants of health, health conditions, and socioeconomic factors. Design weights were applied to enhance the representativeness of the findings (Malter, 2015). Interviewers received standardized training and quality control procedures were implemented to ensure data reliability. Design weights were applied in all analyses using the appropriate weighting procedures in SPSS and R to account for sampling design and non-response. All data are publicly available for scientific use at www.share-project.org.

### Study Variables

#### Outcome Variable: Quality of Life (QoL).

The primary outcome was QoL, assessed with the CASP-12 scale (Wiggins et al., 2008), which measures subjective well-being across four domains: Control, Autonomy, Self-realization, and Pleasure. Each domain includes three items rated on a 4-point Likert scale (1 = never to 4 = often), yielding a total score from 12 to 48, with higher values indicating better QoL. Following previous studies, we classified scores into four categories: 12–34 (low), 35–37 (moderate), 38–39 (high), and 40–48 (very high). The CASP-12 has shown high reliability (Cronbach’s α = 0.84) in older populations (Hyde et al., 2003; Pérez-Rojo et al., 2018).

#### Explanatory Variables: Intrinsic Capacity Domains.

Following WHO’s ICOPE guidelines (WHO, 2019), we considered five domains of intrinsic capacity (IC): locomotion, sensory, cognition, psychological well-being, and vitality (Rojano & Luque et al., 2023; Angelsen et al., 2024). Each domain was dichotomized according to the presence or absence of limitations. Locomotion was measured using SHARE items PH046 (difficulty walking 100 m) and PH047 (difficulty climbing stairs), both recoded into “no difficulty” versus “any limitation.” Additional indicators of functional limitation Activities of Daily Living (ADL) and Instrumental Activities of Daily Living (IADL) were also dichotomized. Sensory function was assessed through perceived vision (PH043) and hearing (PH044) difficulties, each coded as “no difficulty” versus “any impairment.” Cognition was defined as a composite variable integrating episodic memory (CF003, CF006) and spatiotemporal orientation (CF103, CF113, CF114); participants with impairment in either dimension was considered cognitively limited. Psychological well-being was measured with the EURO-D scale (Maskileyson et al., 2021; Portellano-Ortiz et al., 2018), where scores ≥ 4 indicate depression. Vitality was operationalized through Body Mass Index (BMI, PH012, PH013), categorized into underweight (< 18.5), normal (18.5–24.9), overweight (25–29.9), and obesity (≥ 30). For analysis, BMI was dichotomized as “no weight-related issues” (18.5–24.9) versus “weight-related issues” (< 18.5 or ≥ 25). While BMI is an indirect and partial indicator of vitality, it has been widely used in epidemiological research as a proxy for metabolic and nutritional status in large population-based datasets. In the absence of direct measures of energy balance, sarcopenia, or inflammatory biomarkers within SHARE, BMI provides a standardized and comparable operationalization across countries. Nevertheless, we acknowledge that vitality is a multidimensional construct, and future research should incorporate broader physiological indicators to enhance construct validity.

#### Covariates: Social Determinants of Health (SDH) and Sociodemographics.

To contextualize QoL, we included covariates covering SDH, sociodemographic variables, health behaviors, self-perceived health, and comorbidity. SDH included sex, age groups (50–64, 65–74, 75–84, ≥ 85), education (ISCED levels: low, medium, high), financial situation (“ease of making ends meet” and “receipt of financial assistance”), and region of residence (Northern, Continental, Eastern, Southern Europe). Sociodemographic factors included marital status, household composition, and employment status. Health behaviors included physical activity (vigorous activity ≥ once/week vs. less often (Reitlo et al., 2018; Quiroz Mora et al., 2018), alcohol consumption, and tobacco use. Self-perceived health (PH003) was dichotomized into “good” (good/very good) versus “poor” (fair, poor, very poor). Comorbidity was coded as none versus two or more chronic conditions.

All covariates were selected based on prior literature demonstrating their relevance to aging outcomes and quality of life.

### Statistical Analysis

Continuous variables were presented as means and standard deviations, while categorical variables were described using absolute and relative frequencies. Univariate comparisons were conducted using Student’s t-tests for continuous variables and Chi-square tests for categorical variables.

Subsequently, all variables of interest were recoded into dichotomous variables to reflect the presence (1) or absence (0) of functional, cognitive, sensory, or mobility-related limitations, following an adverse event-oriented approach. Each variable was recategorized so that a value of 1 indicated a negative outcome, such as perceived difficulty, chronic condition, or poor performance, based on established cut-off points from the literature or statistical criteria (Salazar Estrada et al., 2019; Salinas-Rodríguez et al., 2022; Berk, 2016). Using this harmonized coding, a composite intrinsic capacity index was calculated by summing the dichotomized variables. This index reflects the accumulation of deficits across domains, where higher scores indicate lower intrinsic capacity (i.e., more domains with limitations), and lower scores represent higher intrinsic capacity. This approach aligns with previous methodologies that assess functional aging through deficit accumulation or frailty indices (Cesari et al., 2018; Ofori-Asenso et al., 2019; Burn et al., 2018), and with studies examining the decline of intrinsic capacity (Rodríguez-Laso et al., 2023; Rojano & Luque et al., 2023). Although the deficit-accumulation strategy resembles approaches used in frailty research, intrinsic capacity differs conceptually in that it focuses on the preservation of functional domains rather than vulnerability to adverse outcomes. While frailty emphasizes risk accumulation, intrinsic capacity emphasizes the level of retained physical and mental capacities. Therefore, the present index should be interpreted as a functional capacity gradient rather than a frailty score. This recoding strategy enabled more effective operationalization of variables for subsequent statistical analyses. Wave 5 data were used for exploratory factor analysis (EFA), while Wave 6 data were employed to assess the predictive validity of the intrinsic capacity instrument.

#### Exploratory Factor Analysis (EFA)

To identify the underlying structure of the constructs assessed, an EFA was conducted using the Weighted Least Squares (WLS) method, appropriate for dichotomous and ordinal data. A polychoric-tetrachoric correlation matrix was used to estimate relationships among categorical variables. The optimal number of factors to retain was determined through Parallel Analysis based on factor analysis, complemented by inspection of the scree plot. To facilitate interpretation, an oblique rotation (Promax) was applied, given the expected correlation among latent factors.

Factor loadings greater than 0.40 were considered significant. Model adequacy was evaluated by examining uniqueness values, and variables with values exceeding 0.70, such as the vitality domain, were excluded from the final model.

#### Network Analysis (NA)

Network analysis was selected because intrinsic capacity is conceptualized as a multidimensional and interactive system rather than a set of independent predictors. Traditional regression approaches assume unidirectional relationships and latent variable structures, whereas network models allow examination of conditional dependencies and the relative centrality of domains within a complex system (Borsboom, 2022). This approach is particularly suitable for aging research, where functional domains interact dynamically and may reinforce or buffer each other over time. Network Analysis was performed using the Huge Estimator approach to explore the structural relationships within intrinsic capacity, minimizing model overfitting and examining how key indicators of mobility, sensory health, mental health, and cognitive function interrelate (Borsboom, 2022). Additionally, Ebiglasso was used to explore directional associations between intrinsic capacity domains and QoL, acknowledging that causal inference cannot be established within the present observational design. This advanced tool enabled modeling of complex, non-linear relationships among variables, facilitating the identification of critical factors with the greatest influence on QoL.

Given the ongoing methodological debate regarding the stability of centrality indices in psychological and health-related networks, the results should be interpreted primarily in terms of overall structural patterns rather than precise centrality rankings. The network was estimated to use regularization procedures to reduce spurious associations and overfitting; however, centrality metrics may be sensitive to sampling variability. Therefore, findings related to node importance are presented as exploratory and hypothesis-generating rather than definitive causal hierarchies.

##### Software Used:

All analyses were conducted using weighted SHARE data and performed with the following software: SPSS version 25 (IBM Corp., Armonk, NY, USA); R version 4.3.0 (R Foundation for Statistical Computing, Vienna, Austria); JASP Stats version 0.19.3. The significance level was set at p < 0.05.

### Ethical Considerations

The Ethics Committee of the Max Planck Society for the Advancement of Science conducted a thorough review of all materials related to the SHARE project, including Wave 5 and the subsequent Wave 6. The committee certified that the research project and its procedures comply with the highest international ethical standards. Strict measures were implemented to ensure the confidentiality and privacy of participants’ data, in accordance with the Declaration of Helsinki and the International Ethical Guidelines for Biomedical Research Involving Human Subjects. Written informed consent was obtained from all participants, who voluntarily agreed to participate during the interviews (Börsch-Supan et al., 2013). Participants were informed about the purpose of the study, data confidentiality, and their right to withdraw at any time without penalty.

Data confidentiality and anonymity were rigorously maintained throughout data collection, processing, and analysis. Personal identifiers were removed prior to data access by researchers, and data were stored securely in compliance with data protection regulations. No individual participant data are reported in this study.

This study did not involve any interventions or clinical trials; therefore, a clinical trial registration number is not applicable.

## Results

### Demographics, healthy lifestyle, and quality of life by sex (Wave 5)

Statistically significant sex differences were found across multiple variables (p < 0.001). Men were slightly older on average than women (64.4 vs. 63.3 years). Women were more represented in the 50–64 age group (54.4% vs. 50.0%). Educational disparities were evident, with 37.0% of women having low education compared to 32.9% of men. Marital status differed substantially, with men more often married or partnered (80.7% vs. 71.2%) and women more frequently widowed (13.4% vs. 4.4%). Economic difficulties were more prevalent among women (31.5% vs. 26.7%). In terms of health behaviors, men reported higher levels of physical activity (57.6% vs. 50.2%), but also greater prevalence of daily alcohol consumption (28.9% vs. 11.5%) and smoking (56.0% vs. 37.1%). Quality of life (QoL), measured by CASP-12, was significantly higher among men than women (38.6 vs. 38.0; p < 0.001). These findings indicate consistent sex differences in socioeconomic conditions and health behaviors. See Table 1 for detailed results.

### Exploratory factor analysis of intrinsic capacity variables

An Exploratory Factor Analysis (EFA) was conducted to uncover the latent structure of intrinsic capacity, using variables related to mobility, cognition, psychological well-being, sensory function, and vitality. Sampling adequacy was acceptable (overall KMO = 0.601; individual values 0.486–0.964), and Bartlett’s test of sphericity (χ^2^ (66) = 138,861, p < 0.001) confirmed the suitability of the data for factor analysis.

Five components with eigenvalues greater than 1 were extracted, jointly explaining 73.05% of the total variance. After Promax (oblique) rotation, the factor structure corresponded to the WHO’s conceptualization of intrinsic capacity, comprising:

1. **Mobility**: locomotor limitations together with ADL and IADL performance,
2. **Psychological state**: depressive symptoms, including fatigue, irritability, and loss of interest.
3. **Cognition**: immediate and delayed word recall, together with orientation tasks.
4. **Sensory function**: self-reported vision and hearing difficulties.
5. **Vitality**: BMI and BMI categories.

The covariance matrix of factor scores indicated negligible covariances (≈ 0), indicating that, although theoretically correlated, the extracted domains showed low empirical overlap in this sample. This structure provides empirical support for the multidimensional nature of intrinsic capacity and validates its operationalization in this dataset. Moreover, the high proportion of explained variance underscores the robustness of the factorial model as a basis for constructing a synthetic index of intrinsic capacity in comparative aging research. See Table 2 for detailed factor loadings and component structure.

### Network analysis of intrinsic capacity structure

**Figure 2:**
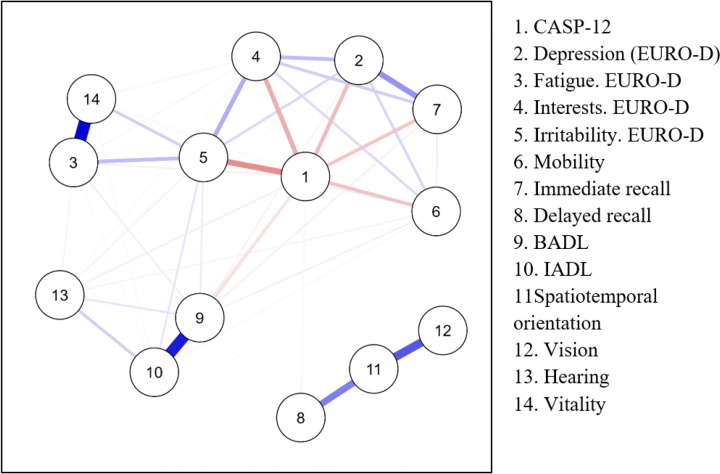
Network analysis of intrinsic capacity domains and quality of life Note: Network of relationships between different domains of intrinsic capacity and quality of life variables in older adults. Nodes represent individual variables, and links indicate the strength and direction of their relationships. Blue links represent positive associations, while red links indicate negative associations. The thickness of the lines reflects the magnitude of the association. Instrumental Activities of Daily Living (IADL); Basic Activities of Daily Living (BADL)

To further examine the structural organization of IC, a Network Analysis (NA) was performed to explore the interactions between IC domains and QoL. The network exhibited a sparsity of 35.9%, indicating that more than one-third of the possible edges were absent, thus supporting the multidimensional nature of IC rather than a single global dimension. Mobility emerged as the most central domain, exhibiting the highest betweenness (1.954), closeness (1.084), strength (1.485), and expected influence (1.590), underscoring its pivotal role in intrinsic capacity. A strong connection was observed between vitality and fatigue, reflecting their close interdependence. Sensory and psychological domains showed weaker connectivity, suggesting more domain-specific effects. The network visualization is provided in Fig. 2.

#### Note

Network of relationships between different domains of intrinsic capacity and quality of life variables in older adults. Nodes represent individual variables, and links indicate the strength and direction of their relationships. Blue links represent positive associations, while red links indicate negative associations. The thickness of the lines reflects the magnitude of the association.

Instrumental Activities of Daily Living (IADL); Basic Activities of Daily Living (BADL)

### Correlations between intrinsic capacity domains and quality of life

Pearson correlation analyses demonstrated that psychological variables had the strongest negative associations with QoL: depressive symptoms (r = − 0.284, p < .001), fatigue (r = − 0.324, p < .001), irritability (r = − 0.230, p < .001), and loss of interest (r = − 0.244, p < .001). Cognitive performance showed weaker but significant negative correlations with CASP-12 scores (r range = − 0.108 to − 0.162, all p < 0.001). Mobility variables exhibited small but significant negative correlations (r range = − 0.019 to − 0.041, p < 0.05). These findings highlight mental health as the strongest direct correlate of QoL, with mobility playing a structurally central role. The full correlation matrix is available in Supplementary Table S2.

### Network analysis of interactions between quality of life and intrinsic capacity domains

A second network analysis including quality of life (CASP-12) and IC domains revealed complete interconnectivity among nodes (dispersion = 0.000). CASP-12 emerged as the most central node, with the highest betweenness (1.789), closeness (0.856), and strength (0.846), but a negative expected influence (− 1.759) indicates that these connections primarily reflected inverse relationships with IC deficits.

**Figure 3:**
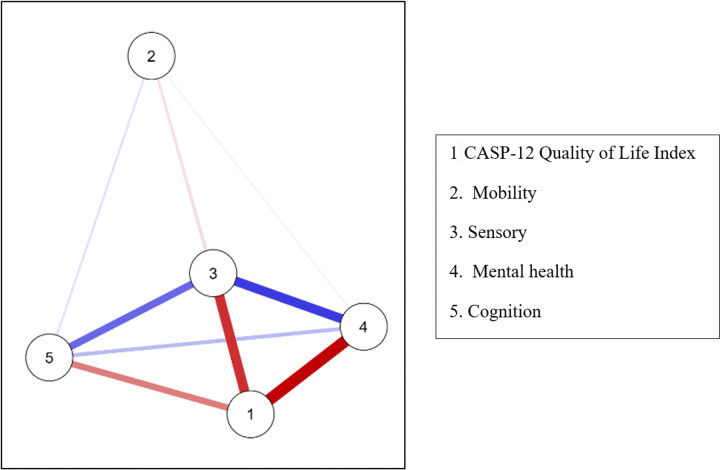
Network Analysis of the Interactions Between Quality of Life and the Domains of Intrinsic Capacity.

Among domains, Mobility showed the lowest connectivity (closeness = − 1.715; strength = − 1.613), whereas Sensory function demonstrated higher integration (closeness = 0.498; strength = 0.650). Clustering measures reinforced this pattern, with CASP and Sensory presenting the highest Onnela coefficients (0.733 and 0.665), while Mobility displayed the weakest integration (Onnela = − 1.712). Psychological and cognitive domains showed moderate cohesion (Zhang index = 0.663 and 0.934). The vitality domain was excluded, as BMI alone did not capture the multidimensional nature of vitality, making it an inadequate proxy in this context. See Fig. 3 for the network graph.

### Sex differences in intrinsic capacity domains

Independent samples t-tests revealed significant sex differences in sensory function, mental health, and cognition. Men had slightly higher sensory scores (M = 0.740) than women (M = 0.697; p = 0.015, d = 0.045). Women exhibited greater emotional distress (mental health domain) than men (M = 1.163 vs. 0.884; p < 0.001, d = − 0.260). Cognitive performance was higher in men (M = 0.609) compared to women (M = 0.518; p < 0.001, d = 0.109). No significant sex difference was found in mobility (p = 0.167). Detailed statistics are provided in Table 3.

#### Total intrinsic capacity by sex and region

ANOVA results showed significant main effects of sex (F (1, 11,485) = 9.575, p = 0.002, η^2^ = 0.0008) and region (F (3, 11,485) = 74.813, p < 0.001, η^2^ = 0.019), as well as a significant sex × region interaction (F (3, 11,485) = 2.825, p = 0.037, η^2^ = 0.0007). Women had slightly higher IC deficit scores overall. Northern Europe exhibited the lowest deficits, while Southern Europe had the highest. Post hoc tests confirmed women in Southern Europe had significantly higher deficits than men in the same region (p = 0.001). See Table 4 for details.

### Socioeconomic and household influences

Intrinsic capacity varied significantly by socioeconomic status and household composition. Employment status had a strong effect (F (6, 11,830) = 168.692, p < 0.001, ω^2^ = 0.078). Self-employed individuals had the lowest IC scores (M = 1.828), while those with medical disabilities had the highest (M = 3.912), significantly exceeding retirees, employees, and the unemployed (all p < 0.001). Detailed comparisons are provided in Supplementary Table S3. Economic difficulty showed a clear gradient (F (3, 11,574) = 146.513, p < 0.001, η^2^ = 0.037). Participants reporting severe financial difficulty had the highest mean IC scores (M = 3.622), whereas those with no difficulty had the lowest (M = 2.291), reflecting better functional capacity (see Table 5).

Household composition influenced outcomes as well. Living with a partner was associated with significantly lower IC scores (F (1, 11,833) = 144.323, p < 0.001, η^2^ = 0.012). Women living alone had the highest deficits (M = 3.252), followed by men living alone (M = 2.879). By contrast, both men (M = 2.482) and women (M = 2.495) living with a partner had lower scores, indicating fewer accumulated deficits. Significant interaction effects (F (1, 11,833) = 14.098, p < 0.001) suggest that cohabitation affects men and women differently (See **Table 6** for further details.)

### Change between waves (2013–2015)

Paired-samples analysis revealed a significant increase in mean IC scores between 2013 (M = 2.628, SD = 2.072) and 2015 (M = 2.766, SD = 2.131), t (11,836) = 7.698, p < 0.001, Cohen’s d = 0.071, indicating a small but measurable decline in intrinsic capacity over two years. Women exhibited greater deficit accumulation, especially in psychological domains. Regional disparities persisted, with Northern Europe showing better outcomes. Older adults aged 75 + experienced sharper declines, particularly in mobility and mental health. Supplementary Table S3 provides complete data.

## Discussion

This study provides robust empirical evidence that intrinsic capacity domains are closely associated with quality-of-life trajectories in older adults across European regions. The findings underscore the multifaceted and multidimensional nature of intrinsic capacity, encompassing interconnected domains such as mobility (including ADL and IADL), cognitive function, mental health, and sensory function. Importantly, these findings offer strong empirical support for the WHO healthy aging framework, which conceptualizes intrinsic capacity as dynamically shaped by environmental and social determinants. The observed sex and regional disparities illustrate that functional aging is not solely biologically driven but deeply embedded within broader welfare and socioeconomic contexts.

These results are consistent with recent research, such as the meta-analysis conducted by Zhou and Ma (2022), which emphasized that intrinsic capacity should be understood as a complex, multidimensional construct rather than a unidimensional entity. The originality of this study lies in the joint application of EFA and Network Analysis to intrinsic capacity, together with a systematic sex- and region-based comparison, which has been scarcely addressed in previous literature.

Through EFA, the study confirmed that intrinsic capacity comprises distinct but interrelated components clustering according to their functional relevance—namely sensory function, cognition, mobility, and mental health. This finding reinforces the need for integrated assessment approach that considers both physical and psychological aspects of aging (Cruz-Peralta & González-Celis, 2023; Leitón Espinoza et al., 2021).

However, the vitality domain, operationalized solely through Body Mass Index (BMI), presented notable limitations. While BMI is a useful epidemiological measure, it fails to capture the complexity of factors influencing intrinsic capacity and quality of life (Leitón Espinoza et al., 2021). In fact, the high uniqueness values observed in the factor analysis, particularly for BMI and its categories, indicated that these variables did not cluster effectively with others, suggesting that BMI alone is insufficient to represents vitality adequately.

Regarding sex differences, the results indicated that women showed a higher accumulation of deficits across several key domains, particularly cognition and mental health. This pattern aligns with previous studies showing that older women tend to face more significant declines in these areas compared to men (Pavez Lizarraga et al., 2023). A combination of biological and sociocultural factors appears to play a critical role in this deterioration, as women’s longer life expectancy increases their exposure to chronic diseases and multimorbidity (Au et al., 2017; San José Laporte, 2012).

The findings of this study reinforce those of Llorens-Ortega et al. (2024), who documented cognitive and physical decline, particularly among older women from socially disadvantaged backgrounds. Conversely, men showed less deterioration in sensory health, reflecting a trend observed in other studies suggesting sex differences in sensory function (Alfonso Silguero et al., 2014). Overall, women’s higher accumulation of deficits, particularly in cognition and psychological domains, highlights persistent sex disparities in functional capacity and quality of life trajectories (Kaur et al., 2024; Concha-Cisternas et al., 2021; Díaz-Alonso et al., 2021).

Mental health, particularly depression, emerged as a key domain with significantly higher prevalence among women over time. Depression is known to accelerate functional decline, particularly in females. Previous research has emphasized the central role of psychological well-being in shaping perceived quality of life, identifying mental health as a key determinant of subjective well-being (Kim, 2025). Our findings are consistent with studies documenting increased depressive symptoms among older women, attributable to a combination of biological factors and the accumulation of psychosocial stressors throughout life (Jalali et al., 2024; Portellano-Ortiz et al., 2018).

Moreover, factors such as social isolation, lack of emotional support, and greater caregiving burden exacerbate depression’s negative impact on older women’s quality of life (Courtin & Knapp, 2017; Lee et al., 2022). These results reinforce the urgent need for sex-sensitive public health policies, including enhanced access to community-based mental health services, systematic depression screening in primary care, and tailored psychosocial support for vulnerable women.

The inclusion of social determinants of health (SDH) in this study was essential to understanding how factors such as educational level, marital status, and economic situation are consistently associated with functional decline and quality of life in older adults (Bielderman et al., 2015; Steptoe & Zaninotto, 2020). Across the European regions analyzed, women in socially and economically vulnerable situations exhibited greater deterioration in mental health, a significant increase in chronic conditions, and more pronounced economic decline. This pattern aligns with recent studies showing that social inequalities disproportionately affect older women, particularly in contexts of poverty or limited access to healthcare (Bacigalupe et al., 2022; Spiers et al., 2022). Similarly, previous research has shown that social participation and broader social conditions play a critical role in shaping well-being and functional trajectories among older adults (Oshio et al., 2024).

From a policy perspective, these findings highlight the importance of reducing socioeconomic inequalities to mitigate IC decline. Interventions should include income protection for older adults, universal access to healthcare and long-term care services, and programs that address social isolation and support for those living alone (Lu et al., 2025). Importantly, harmonizing social protection policies across European regions could reduce disparities observed between Northern and Southern/Eastern Europe.

The network analysis elucidated the underlying structure of intrinsic capacity, revealing mobility as a structurally central domain, while psychological and sensory domains showed stronger direct associations with quality of life. Although mobility and cognition are important domains, their impact on quality of life appears to be more modest compared to physical and mental health. This finding aligns with previous studies demonstrating that physical and mental health are key determinants of quality of life in older adults (Geigl et al., 2023; Gutiérrez-Robledo et al., 2021; Sugimoto et al., 2022). These results suggest that interventions prioritizing mental well-being and physical resilience may yield the most significant improvements in overall QoL.

### Strengths and limitations

Among the strengths of this study is the use of a large representative sample from 13 European countries, a short-term longitudinal and comparative perspective across two consecutive waves. Additionally, the inclusion of social determinants of health enriched the analysis, allowing for the identification of socioeconomic and geographic disparities.

However, the study also presents several limitations. The use of self-reported data may introduce bias, particularly in domains such as mental health and mobility. Second, although longitudinal data were used, the observational design does not allow full disentanglement of bidirectional relationships between intrinsic capacity domains and quality of life. Psychological well-being and QoL may mutually influence each other over time, and causal inferences should therefore be interpreted cautiously. Furthermore, the vitality domain’s operationalization via BMI is limited as this measure does not fully capture the multidimensional nature of vitality. Future studies should incorporate additional indicators to provide a more accurate representation of this domain. The relatively short follow-up period (2 years) may underestimate longer-term intrinsic capacity trajectories. Additionally, although regularized network models reduce overfitting, the stability of centrality indices may vary across samples, and replication in independent cohorts is warranted. Residual confounding cannot be fully excluded despite adjustment for multiple covariates.

Moreover, variability in health policy implementation across European countries may have influenced the results. This suggests that future research should include longer longitudinal data and more diverse samples to better assess changes in quality of life and intrinsic capacity over time.

### Public health implications

The findings of this study have direct implications for public health policy. Addressing disparities in intrinsic capacity and quality of life requires integrated strategies at both the individual and structural levels:

1. **Mental health interventions**: Implement systematic depression screening in primary care, expand psychological services tailored for older adults, and adopt sex-sensitive approaches that consider caregiving burdens and social isolation.
2. **Mobility promotion**: Develop community-based exercise programs, implement fall prevention strategies, and promote urban planning that fosters age-friendly environments to support physical resilience.
3. **Nutritional and vitality programs**: Design initiatives that go beyond Body Mass Index (BMI) to address malnutrition, frailty, and energy balance, incorporating comprehensive assessments of vitality.
4. **Reducing regional disparities**: Advocate for EU-level policies that harmonize access to health and long-term care services, with targeted support for Southern and Eastern European regions where deficits are more pronounced.
5. **Social and economic protection**: Strengthen financial security through pensions and assistance programs, promote social participation, and provide support for individuals living alone, especially widowed or divorced women.

By integrating clinical, behavioral, and policy-oriented interventions, these measures could help reduce sex and regional disparities, ultimately promoting equitable and healthy aging across Europe.

Conceptually, this study contributes to bridge the gap between the WHO intrinsic capacity framework and the social determinants of health perspective. It demonstrates that intrinsic capacity domains are not only biologically grounded but also socially patterned across sex and welfare regimes. Therefore, healthy aging must be understood within broader structural contexts, recognizing intrinsic capacity as a socially embedded functional construct shaped by life-course inequalities.

## Conclusions

This study provides empirical evidence that intrinsic capacity (IC) is a multidimensional construct consistently associated with quality of life (QoL) in older adults across Europe. Sex-related differences were observed, with women showing higher accumulation of deficits particularly in cognition and mental health domains, while regional patterns showed that Southern and Eastern Europe accumulated more deficits compared to Northern regions. Social determinants such as education and economic status were also associated with these disparities.

Together, these findings highlight the need for targeted actions addressing sex and regional inequalities in aging. Strengthening mental health care and mobility support may represent promising strategic areas for preserving intrinsic capacity and improving quality of life in older adults.

## Supplementary Material

Supplementary Files

This is a list of supplementary files associated with this preprint. Click to download.

• SupplementaryMaterial..docx

• Table3To6.docx

## Figures and Tables

**Table 1:** Sociodemographic characteristics, healthy lifestyle, and quality of life by Sex (Wave 5)

Variable %	Women (N = 6236)	Men (N = 5257)	Total (N = 11493)	p-value
**Demographic Data:**						
Age (SD)	63.3 (10.2)	64.4 (9.36)	63.8 (9.83)	<0.001
**Age Group:**				<0.001
50–64	3392 (54.4%)	2629 (50.0%)	6021 (52.4%)	
65–74	1753 (28.1%)	1668 (31.7%)	3421 (29.8%)	
75–84	885 (14.2%)	819 (15.6%)	1704 (14.8%)	
85+	206 (3.30%)	141 (2.68%)	347 (3.02%)	
**Educational Level:**				<0.001
Low	2310 (37.0%)	1727 (32.9%)	4037 (35.1%)	
Medium	2326 (37.3%)	2037 (38.7%)	4363 (38.0%)	
High	1600 (25.7%)	1493 (28.4%)	3093 (26.9%)	
**Marital Status:**				<0.001
Married or registered partner	4443 (71.2%)	4241 (80.7%)	8684 (75.6%)	
Divorced or separated	638 (10.2%)	432 (8.22%)	1070 (9.31%)	
Single	319 (5.12%)	350 (6.66%)	669 (5.82%)	
Widowed	836 (13.4%)	234 (4.45%)	1070 (9.31%)	
**Able to make ends meet**	4337 (69.5%)	3853 (73.3%)	8190 (71.3%)	<0.001
**Received help from others (outside the household)**	1174 (18.8%)	833 (15.8%)	2007 (17.5%)	<0.001
**Healthy Lifestyle:**				
Physical Activity	3132 (50.2%)	3027 (57.6%)	6159 (53.6%)	<0.001
**Daily Alcohol Consumption:**				<0.001
None/1–2 times per month	3801 (61.0%)	1950 (37.1%)	5751 (50.0%)	
1–4 days per week	1719 (27.6%)	1790 (34.0%)	3509 (30.5%)	
Almost daily	716 (11.5%)	1517 (28.9%)	2233 (19.4%)	
Daily Smoking	2314 (37.1%)	2944 (56.0%)	5258 (45.7%)	<0.001
**Quality of Life**: CASP (SD)	38.0 (6.37)	38.6 (6.17)	38.3 (6.29)	<0.001

Note: Statistical tests used: ANOVA for continuous variables and Chi-square for categorical variables.

**Table 2:** Factor analysis of intrinsic capacity variables

Variable	Factor 1 (Mobility)	Factor 2 (Psychological State)	Factor 3 (Cognition)	Factor 4 (Sensory Function)	Factor 5 (Vitality)	Uniqueness
Mobility problems	0.865					0.325
ADL	0.867					0.385
IADL	0.787					0.355
Depression scale (EuroD)		0.486				0.437
Immediate word recall			0.928			0.448
Delayed word recall			0.928			0.489
Orientation in time and space			0.676			0.565
Vision difficulty				0.908		0.443
Hearing difficulty				0.854		0.451
BMI					0.961	0.934
BMI categories					0.964	0.934

Note: Rotated factor loadings are shown (Promax, oblique rotation). Each variable is primarily associated with a single domain of intrinsic capacity as defined by the WHO: mobility, psychological state, cognition, sensory function, and vitality. Activities of Daily Living (ADL). Instrumental Activities of Daily Living (IADL); BMI: Body Mass Index

## Data Availability

The data that support the findings of this study are available from the Survey of Health, Ageing and Retirement in Europe (SHARE). SHARE data are publicly available for scientific research upon registration and approval at: https://share-eric.eu/data/data-access The analyses in this study are based on SHARE Waves 5 (2013) and 6 (2015).

## Abbreviations

ADL: Activities of Daily Living
BMI: Body Mass Index
CASP-12: Control, Autonomy, Self-realization, Pleasure scale
EFA: Exploratory Factor Analysis
EURO-D: European Depression Scale
IADL: Instrumental Activities of Daily Living
IC: Intrinsic capacity
NA: Network Analysis
QoL: Quality of life
SDH: Social Determinants of Health
SHARE: Survey of Health, Ageing and Retirement in Europe
